# Assessment of novel technologies in healthcare - off-label use of drugs and the ethics of implementation and distribution of COVID-19 vaccines

**DOI:** 10.31744/einstein_journal/2021ED6840

**Published:** 2021-12-20

**Authors:** Vivian Iida Avelino-Silva, Mario Thadeu Leme de Barros, Class 8 FICSAE Working Group

**Affiliations:** 1 Faculdade Israelita de Ciências da Saúde Albert Einstein Hospital Israelita Albert Einstein São Paulo SP Brazil Faculdade Israelita de Ciências da Saúde Albert Einstein, Hospital Israelita Albert Einstein, São Paulo, SP, Brazil.

New health technologies, such as new medications, products, equipment, and vaccines, are constantly produced. The traditional development of new health technologies is a lenghty and high-cost process, including pre-clinical development phases (*in vitro* and animal model studies) and clinical trials, traditionally divided into phases 1, 2 and 3. The registry of novel technologies must be required at the National Health Surveillance Agency (Anvisa - *Agência Nacional de Vigilância Sanitária*), only after the conclusion of clinical trials. The agency has a technical team responsible for evaluating and determining whether or not the novel technology can be introduced in the Brazilian market.^(1)^

Nonetheless, the effective incorporation of novel technologies in clinical practice also depends on demonstrating that they will in fact benefit both patients and the society. Health Technology Assessment (HTA) consists of a multistep analyses based on scientific evidence, implemented as an essential criterion for decisions about the incorporation of novel health technologies in the context of the Public Health System (SUS - *Sistema Único de Saúde*), since the ratification of Act 12,401, of April 28^th^, 2011.^(2)^

Health Technology Assessment is a decision-making tool, based on unbiased and transparent deliberations regarding the following aspects:

- Clinical: evaluation of safety, efficacy, effectiveness, clinical indications, and target population, considering the burden of the disease and its social impacts.
- Economic: evaluation of economic studies (studies on cost, cost-effectiveness, cost-utility, and cost-benefit) and impact on budget.
- Patient-related: evaluation of characteristics and social impact of the disease, importance and benefits of technologies already implemented, patient demands that are currently overlooked, convenience, public perception and acceptance of the new technology, and ethical issues related to the implementation of the novel technology.
- Organizational: evaluation of the feasibility for widespread use, capacity-building of professionals, optimized allocation of resources, monitoring of results, and sustainability of the new health technology.

The HTA process is employed in several countries with similar principles, but adapted to the local context, as a tool to support decisions made by health managers. In Brazil, the National Committee for Health Technology Incorporation in the SUS (CONITEC - *Comissão Nacional de Incorporação de Tecnologias),* a technical agency of health policy, uses the HTA as a strategy to help the Ministry of Health in evaluating the implementation of novel technologies at SUS.^(3,4)^

The participation of the civil society in the HTA process is anticipated and encouraged, aiming to add pieces of information about the impacts of the disease, limitations imposed to quality of life, and expectations concerning the benefits associated with new technologies as compared to available interventions. Information gathered from patients and caregivers improve the overall understanding and implementation of novel technologies, considering the health-related preferences of the target population. It is particularly important to have the civil society participating in the discussion about HTA. There are four main mechanisms for including public participation in discussions concerning the incorporation of new health technologies, as follows: public consultations; court hearings; surveys; and participation in plenary sessions. It is also worth mentioning that the engagement of the civil society can also occur by the mere access to information, research, analyses, and recommendations published by CONITEC and available for the general public.^(2)^

## Assessment of new health technologies in the context of off-label use and repurposing of drugs for treatment and prevention of COVID-19

Some medications are used for purposes that are not described in the package insert; therefore, they are not analyzed in controlled studies, neither submitted to evaluation by regulatory processes, such as HTA, or official agencies, such as CONITEC, the National Health Agency (ANS - *Agência Nacional de Saúde*) and Anvisa. This practice is generally known as off-label use.^(5)^ The off-label use of medications or other medical technologies must be differentiated from repurposed or repositioned use, in which medications that are already approved are submitted to a rigorous and systematic process of analysis, aiming to identify compounds that might be applied to other conditions, such as emerging or rare diseases, for which no specific treatment is available.^(6)^ Drug repurposing has advantages relatively to the traditional development of new medicines, since repurposed drugs have often undergone safety studies, and can be implemented faster, with lower financial investments. The initial identification of candidate compounds for repurposing can be conducted by experimental or computational methods, which identify molecules more likely to act in targets of interest. Another option is to select candidate compounds that have phenotypical or functional characteristics similar to the drugs already used for that specific purpose.^(6)^

While repurposing of drugs implies the demonstration of efficacy in clinical trials, off-label use can be indicated by a physician, who simply believes the patient will benefit from that medication. In the case of medicines sold over the counter, off-label use can be decided by the patients themselves, not requiring medical prescriptions. Off-label use of medications may imply risks not only for patients but also for physicians, due to the lack of clinical studies or approval of the new purpose of the drug by Anvisa, leaving them with no legal support. Moreover, off-label prescription exempts the pharmaceutical industry from legal and judicial responsibilities if adverse reactions occur, since this use is not described in the package insert.^(7)^

The search for therapeutic interventions for the coronavirus disease 2019 (COVID-19) has been non-stop since the beginning of the pandemic. Several medications have been used off-label for treatment and prevention of COVID-19, based on reports of effect against the severe acute respiratory syndrome coronavirus 2 (SARS-CoV-2) in *in vitro* studies, or human studies with major methodological limitations.^(8-11)^ A few months after the pandemic began, controlled clinical trials were published,^(12-18)^ subsidizing the publication of guidelines that contraindicated the use of a large number of these medications.^(19-21)^ However, many of these drugs are still being prescribed, despite the evidence of futility from several studies.^(22)^ The widespread use of such medications has several harmful consequences, including the occurrence of adverse events;^(23)^a disproportionate consumption leading to supply shortage;^(24,25)^ higher prices in the market;^(26)^ and development of antimicrobial resistance, in the case of extensive use of antibiotics for such purpose.^(27)^ Moreover, the benefits of HTA, including technical, economic, and operational deliberations, and the participation of the civil society, are not applied for off-label use of medications. Finally, persisting on the off-label prescription of medications for treatment and prevention of COVID-19, even after the demonstration of their inefficacy, no longer stands as an exercise of medical autonomy, but could be legally characterized as medical error.^(7)^

## Applications of new health technology assessment: ethical aspects of the implementation of COVID-19 vaccines

Among the new health technologies developed for COVID-19, vaccines are arguably the most important strategy to control the pandemic, and have been implemented in Brazil based on a HTA process.^(28)^ Despite the current robust evidence on safety,^(29-36)^ efficacy,^(31,32,35,37,38)^ effectiveness^(30,33,39,40)^ and cost-effectiveness^(41,42)^of COVID-19 vaccines, some operational and ethical aspects related to the incorporation of these new health technologies, such as distribution strategies and priority groups, should be considered with caution.^(43)^

Although COVID-19 vaccines have been rapidly developed by different manufacturers, the number of doses is still much beyond the required volume to immunize the world population. It has been necessary to allocate resources giving priority to certain places and population groups.^(43)^Furthermore, compliance to vaccine recommendations has not been uniform in the population. Hence, implementing COVID-19 vaccines has raised important ethical discussions.^(44)^

Access to COVID-19 vaccines has been effective and quick in high-income countries when compared to more deprived regions.^(43)^ The United States, Canada, United Kingdom, and Israel, were the first countries to reserve large batches of vaccines, taking advantage of their higher purchasing and negotiation capacity.^(43,45)^ According to this market-based system, low-income countries have had access to a reduced number of COVID-19 vaccine batches. According to the World Health Organization (WHO), ten countries concentrate approximately 75% of all COVID-19 vaccines worldwide.^(46)^ Tedros Adhanom, general director of WHO, declared “the world is on the brink of a catastrophic moral failure – and the price of this failure will be paid with lives and livelihoods in the world’s poorest countries”.^(47)^

This inequity in the distribution of COVID-19 vaccines has relevant practical implications. While there is no international mobilization for the distribution of doses to more deprived countries, with more cases of the disease and excessive number of avoidable deaths, one could anticipate the persistence of the pandemic, due to the emergence and spread of new variants of the virus.^(48)^ In addition, countries with delayed implementation of the vaccine will also suffer from greater impact to the health system and increased need of non-pharmacological measures, such as physical distancing, trade restrictions, and closing of schools, with repercussions that amplify the social and economic abyss in relation to richer countries.^(43)^

Another ethical aspect of implementing COVID-19 vaccines has been the priority given to certain population groups in the vaccination strategy. Ethical considerations include the principles of Utility (allocation of resources aiming to maximize benefits and reduce drawbacks); Justice (prioritizing individuals and communities who are underprivileged or at higher risk of negative outcomes); as well as giving priority to workers directly involved in patient care.^(44,49)^In Brazil, the priority groups, as outlined in the National Operationalization Plan for COVID-19 Vaccination,^(50)^ included healthcare workers, indigenous and *quilombolas*, older persons (in descending age order) and, more recently, people with underlying medical conditions associated to poorer outcomes in COVID-19. Although apparently suitable, we believe the vaccination strategy should not aggravate the social inequalities in our society, and highlight several ethical issues related to priority groups for COVID-19 vaccines in Brazil.

Healthcare workers included those with technical training or university degree; however, unskilled and less qualified workers or those with no formal employment, such as maintenance and security workers, were not uniformly included in the priority group for vaccination, despite delivering direct care to patients. This fact underlined and aggravated inequalities, since it is a benefit that once again excluded the victims of precarious work conditions.

Although indigenous and *quilombolas* have been included as priority groups for vaccination, marginalized and socially excluded populations may distrust the government actions, due to the historic lack of support by the State, or even the exploitation experience;^(51,52)^ the estimated vaccine coverage with two doses among indigenous peoples in Brazil varied between 42% and 93%.^(53)^ Furthermore, the paucity of actions informing about the safety and importance of vaccination for these populations may have had a significant impact in the low vaccine coverage in these groups.^(54)^

Setting priority to older adults based on a descending age order places a paramount importance on age. In fact, this criteria could be better characterized by other factors, such as frailty.^(55)^ Moreover, in Brazil and other countries, ageing is a privilege of white individuals of more favored social brackets.^(56)^

Several studies have demonstrated that black and *pardo* people are at a greater risk of death following COVID-19,^(57-59)^ but the vaccination plan did not give priority to individuals according to race/skin color. Additionally, individuals with worse housing and sanitation conditions, and less likely to comply with the recommendations of physical distancing, were not prioritized in the vaccination strategy.

Finally, in view of the growing movement of vaccine hesitancy,^(43)^the implementation of mandatory vaccination for COVID-19 has been discussed in some contexts.^(60-63)^ Ethical considerations concerning this debate include the principles of autonomy and individual freedom, as opposed to solidarity and collective well-being. However, one of the most important characteristics of fundamental rights is that no single right is absolute. In other words, refusing a vaccine is an individual right, but this action may cause losses to society by hindering or delaying collective benefits, such as herd immunity and reductions of overloads in health facilities, or by posing risks to susceptible persons. In a pandemic, it must be understood that the individual and collective interests are mixed, since the individual will be protected only when the collectivity is safe. The general understanding of rights and duties as an exercise of civility must be reconsidered to effectively support the social interest as an extent of the individual interest. Solidarity is relevant and must receive cooperative consideration. No one is safe in an epidemic while being alone. Protection or prevention actions must have a collective nature. We must promote a balance between freedom and social solidarity.

Policies of compulsory vaccination often accept exemptions (for instance, medical contraindications or religious restrictions) and do not involve direct punishment or criminal implications against individuals; however, these policies may enforce restrictions in activities, such as attending schools, carrying out certain professional activities^(64)^ or traveling. Before implementing compulsory vaccines, it is essential to employ all possible strategies of information and persuasion; to guarantee enough supplies for vaccination; and to address if the compulsory use will be proportionally corroborated by the expected benefit (that is, the number of individuals vaccinated with this strategy justifies the achieved collective good). It is paramount to consider that policies of compulsory use can trigger negative reactions, hindering the trust of the population in government actions, and even the compliance with other public health measures.^(60)^

The COVID-19 pandemic shed light on the importance of assessing new health technologies as a strategy to implement medications and vaccines in clinical practice and at SUS. In a pandemic characterized by a high burden of cases and deaths, as well as intense social impact, the ethical perspective is crucial and must guide the evaluation of clinical, economic, organizational, and social aspects of HTA. Any political measure is doomed to failure if scientific and health processes are not respected and understood, and this fact emphasizes the importance of assessing new health technologies. Solidarity must be understood as a shared practice that allows each individual to assume costs and tasks, facing challenges that are relevant to the whole society . It is worth mentioning that individual health depends on the cooperation in collective health, based on transparency and trust. We must give room to the rebirth of a society guided by “us” rather than “me”.
