# Supplementary material for: Assessment of novel technologies in healthcare - off-label use of drugs and the ethics of implementation and distribution of COVID-19 vaccines
Source: Einstein (Sao Paulo). 2021 Dec 20;19:eED6840. doi: 10.31744/einstein_journal/2021ED6840 (PMC8693880; doi:10.31744/einstein_journal/2021ED6840)
Supplement: Translation

# Translation: Avaliação de novas tecnologias em saúde – o uso off label de fármacos e a ética do uso e da distribuição de vacinas contra a COVID-19

**Authors:** Vivian Iida Avelino-Silva, Mario Thadeu Leme de Barros, Turma 8 FICSAE Grupo de Trabalho

**Affiliations:** 1 Brasil Faculdade Israelita de Ciências da Saúde Albert Einstein, Hospital Israelita Albert Einstein, São Paulo, SP, Brasil.

Novas tecnologias em saúde, tais como novos medicamentos, produtos, equipamentos e vacinas, são constantemente produzidas. O desenvolvimento tradicional de novas tecnologias em saúde é um processo demorado e de alto custo, incluindo as fases de desenvolvimento pré-clínico (estudos *in vitro* e em modelos animais) e estudos clínicos de fase 1, 2 e 3. Somente após a conclusão dos estudos clínicos, o registro de novas tecnologias pode ser solicitado junto à Agência Nacional de Vigilância Sanitária (Anvisa), que dispõe de uma equipe técnica responsável por avaliar e autorizar ou não a entrada da nova tecnologia no mercado brasileiro.^(1)^

Entretanto, a efetiva incorporação das novas tecnologias na prática clínica depende da demonstração de que elas, de fato, representam um ganho para pacientes e para a sociedade. A Avaliação de Tecnologias em Saúde (ATS) consiste em um conjunto de análises baseadas em evidências científicas, implementadas como critério indispensável para a tomada de decisões sobre a incorporação de novas tecnologias em saúde no contexto do Sistema Único de Saúde (SUS) desde a homologação da lei 12.401 de 28 de abril de 2011.^(2)^

A ATS é uma ferramenta de tomada de decisões, construída a partir de deliberações imparciais e transparentes sobre os seguintes aspectos:

- Clínico: avaliação de segurança, eficácia, efetividade, indicações clínicas e população beneficiada, levando em consideração a carga da doença e os impactos sociais.
- Econômico: avaliação em estudos econômicos (estudos de custo, custo-efetividade, custo-utilidade e custo-benefício) e impacto orçamentário.
- Do paciente: avaliação das características e do impacto social da doença, importância e benefício de tecnologias já implementadas, demandas ainda não contempladas, aceitabilidade, conveniência e aspectos éticos relacionados à implementação da nova tecnologia.
- Organizacional: avaliação da factibilidade de difusão, capacitação de profissionais, uso otimizado de recursos, monitoramento de resultados e sustentabilidade da nova tecnologia em saúde.

O processo da ATS é utilizado em vários países com princípios análogos, mas adaptados ao contexto local, sendo aplicado nas decisões por gestores de saúde. No Brasil, a Comissão Nacional de Incorporação de Tecnologias no SUS (CONITEC), órgão técnico da política de saúde, utiliza a ATS como estratégia para auxiliar o Ministério da Saúde a avaliar a incorporação no SUS de novas tecnologias.^(3,4)^

A participação da sociedade civil no processo da ATS é prevista e encorajada, com o intuito de incorporar informações sobre os impactos da doença, limitações impostas à qualidade de vida e expectativas em relação aos benefícios associados a novas tecnologias em comparação a intervenções disponíveis. Informações obtidas a partir da perspectiva de pacientes e cuidadores melhoram a compreensão e a implementação das novas tecnologias, tendo em vista as preferências em saúde do próprio público-alvo. É muito importante a participação da sociedade civil na discussão da ATS. Os mecanismos de participação da sociedade na construção das possibilidades de incorporação de tecnologias em saúde são, principalmente, quatro: as consultas públicas, as audiências públicas, as enquetes e a participação em plenárias. Vale ressaltar também que a participação da sociedade, para além destes mecanismos participativos, se dá, muitas vezes, pelo simples acesso a informações, pesquisas, análises e recomendações publicadas pela CONITEC para o público.^(2)^

## Avaliação de novas tecnologias em saúde no contexto do uso off label e redirecionamento de fármacos para o tratamento e a prevenção da COVID-19

Algumas medicações são utilizadas para finalidades não previstas na bula e, assim, não são analisadas em estudos controlados e nem submetidas à avaliação por processos regulatórios, como a ATS, ou a órgãos oficiais, como a CONITEC, a Agência Nacional de Saúde (ANS) e a Anvisa. Esse tipo de uso é habitualmente conhecido como *off label.*^(5)^ O uso *off label* de medicamentos ou outras tecnologias médicas deve ser discriminado do uso reposicionado ou redirecionado, no qual medicações já aprovadas são submetidas a um processo rigoroso e sistemático de análise, com o objetivo de identificar compostos que possam ser aplicados a outras finalidades, como doenças emergentes ou raras, que não possuem tratamento específico.^(6)^ O redirecionamento de fármacos possui vantagens em relação ao desenvolvimento tradicional de novas medicações, uma vez que as drogas redirecionadas habitualmente já passaram por estudos de segurança e podem ser implementadas de forma mais rápida e com menor investimento financeiro. A identificação inicial de compostos candidatos para redirecionamento pode ser realizada por meio de métodos experimentais ou computacionais, que identificam moléculas com maior probabilidade de ação em alvos de interesse, ou selecionam compostos candidatos que possuam características fenotípicas ou funcionais semelhantes a drogas já utilizadas para a finalidade em questão.^(6)^

Enquanto o redirecionamento de drogas implica na realização de estudos clínicos de eficácia, o uso *off label* pode ser indicado por um profissional médico que simplesmente acredite que o paciente se beneficiará daquela medicação. No caso de medicações comercializadas sem receita médica, o uso *off label* pode ser feito pelo próprio paciente de forma não controlada. Dessa forma, o uso *off label* de medicações pode implicar em riscos para o paciente e para o médico, pela ausência de estudos clínicos ou da aprovação da nova finalidade do fármaco pela Anvisa, deixando-os sem respaldo jurídico. Além disso, prescrição *off label* isenta a empresa farmacêutica de responsabilidades legais e jurídicas caso ocorram reações adversas, já que esse uso não está descrito na bula.^(7)^

A busca por intervenções terapêuticas para a doença pelo coronavírus 2019 (COVID-19) tem sido incessante desde o início da pandemia. Diversas medicações vêm sendo utilizadas na condição *off label* para o tratamento e a prevenção da COVID-19, motivadas por relatos de efeito contra o coronavírus da síndrome respiratória aguda grave 2 (SARS-CoV-2) em estudos *in vitro* ou em estudos humanos com importantes limitações metodológicas.^(8-11)^ Estudos clínicos controlados foram posteriormente publicados,^(12-18)^ subsidiando a divulgação de diretrizes que contraindicam o uso de grande parte dessas medicações.^(19-21)^ No entanto, muitos desses fármacos continuam sendo prescritos em detrimento da existência de estudos comprovando sua futilidade.^(22)^ O uso indiscriminado de tais medicações tem diversas consequências danosas, incluindo a ocorrência de eventos adversos,^(23)^o consumo desproporcional levando à escassez dos produtos,^(24,25)^ o encarecimento no mercado^(26)^ e o desenvolvimento de resistência antimicrobiana, no caso do uso extensivo de antibióticos para essa finalidade.^(27)^ Benefícios da ATS, incluindo deliberações técnicas, econômicas, operacionais e a participação da sociedade civil, deixam de ser aplicados à situação de uso *off label* de medicações. Por fim, a persistência do uso *off label* de medicações para o tratamento e a prevenção da COVID-19, mesmo após a demonstração de ineficácia, pode legalmente caracterizar não mais a autonomia médica, mas sim o erro médico.^(7)^

## Aplicações da avaliação de novas tecnologias em saúde: aspectos éticos da implementação das vacinas da COVID-19

Dentre as novas tecnologias em saúde desenvolvidas para a COVID-19, as vacinas constituem a mais importante estratégia para o controle da pandemia, e sua implementação vem sendo instituída no Brasil juntamente do processo de ATS.^(28)^ Apesar das evidências robustas atualmente disponíveis acerca da segurança,^(29-36)^ da eficácia,^(31,32,35,37,38)^ da efetividade^(30,33,39,40)^ e do custo-efetividade^(41,42)^ das vacinas da COVID-19, aspectos operacionais e éticos relacionados à incorporação dessas novas tecnologias em saúde, como a forma de implementação e os grupos priorizados, ainda devem ser cuidadosamente considerados.^(43)^

As vacinas da COVID-19 vêm sendo desenvolvidas de maneira acelerada por diferentes fabricantes, porém a produção de doses encontra-se ainda muito aquém do volume necessário para imunizar a população mundial, sendo necessário alocar recursos com priorização de determinados locais e grupos populacionais.^(43)^Além disso, a adesão às recomendações da vacina não é uniforme na população. Dessa forma, a implementação de vacinas da COVID-19 vem suscitando importantes discussões éticas.^(44)^

O acesso às vacinas da COVID-19 ocorre de forma mais efetiva e rápida em países de alto nível socioeconômico quando comparados a países mais carentes.^(43)^ Países como os Estados Unidos, Canadá, Reino Unido e Israel anteciparam-se na reserva de grandes lotes de vacinas, tomando proveito de seu maior poder de compra e maior capacidade de negociação.^(43,45)^ Seguindo essa lógica de mercado, países de menor nível socioeconômico vêm tendo acesso a um número reduzido de lotes de vacinas da COVID-19. Segundo a Organização Mundial da Saúde (OMS), dez países concentram cerca de 75% de todas as vacinas contra a COVID-19 no mundo todo.^(46)^ Tedros Adhanom, diretor-geral da OMS, declarou que “o mundo está à beira de um fracasso moral catastrófico, e o preço desse fracasso será pago com as vidas e meios de subsistência dos países mais pobres”.^(47)^

Essa inequidade na distribuição das vacinas da COVID-19 tem implicações práticas importantes. Enquanto não houver mobilização internacional para a distribuição das doses aos países mais carentes e com maior número de casos, além do excesso de mortes evitáveis, podemos também antecipar a persistência da pandemia, devido ao surgimento e à circulação de novas variantes do vírus.^(48)^ Além disso, países com menor velocidade de implementação da vacinação sofrem também com maior impacto sobre o sistema de saúde e maior necessidade de medidas não farmacológicas, como distanciamento físico, restrições comerciais e fechamento de escolas, com repercussões que ampliam o abismo social e econômico em relação a países mais favorecidos.^(43)^

Outro aspecto ético da implementação das vacinas da COVID-19 diz respeito à priorização de determinados segmentos populacionais na estratégia de vacinação. Considerações éticas envolvidas na priorização das doses de vacinas da COVID-19 incluem os princípios da utilidade (alocação de recursos conforme a capacidade de maximizar os benefícios e reduzir os prejuízos) e da justiça (priorização de indivíduos e comunidades menos favorecidos ou com maior risco de desfechos negativos) e a priorização de trabalhadores envolvidos com o cuidado direto a pacientes.^(44,49)^No Brasil, os grupos considerados prioritários, conforme descrito no Plano Nacional de Operacionalização da Vacinação contra COVID-19,^(50)^ incluíram profissionais de saúde, indígenas e quilombolas, idosos (por ordem decrescente de idade) e, mais recentemente, pessoas com comorbidades associadas a pior prognóstico na COVID-19. Embora aparentemente adequadas, destacamos que a estratégia de vacinação não pode agravar as desigualdades sociais em nossa sociedade e podemos apontar diversos questionamentos éticos relacionados à priorização de vacinas no Brasil.

Foram contemplados, como profissionais de saúde, os trabalhadores com formação técnica ou que possuem curso superior completo; entretanto, trabalhadores sem qualificação ou vínculo empregatício formal com as unidades de saúde, como profissionais de limpeza e segurança, não foram uniformemente incluídos no grupo prioritário de vacinação, apesar do atendimento direto a pacientes. Isso reforça e agrava as desigualdades, por se tratar de um benefício que exclui, mais uma vez, as vítimas da precarização laboral.

Embora indígenas e quilombolas tenham sido incluídos na prioridade para vacinação, populações invisibilizadas e socialmente excluídas podem apresentar menor confiança em ações governamentais, motivada pela histórica escassez de suporte do estado ou, ainda, por antecedentes de exploração;^(51,52)^ a estimativa de cobertura vacinal com duas doses entre povos indígenas no Brasil variou entre 42% e 93%,^(53)^ e a carência de ações para o esclarecimento sobre a segurança e a importância da vacinação para essas populações pode ter tido impacto significativo na baixa cobertura vacinal em alguns povos.^(54)^

A priorização de pessoas idosas por ordem decrescente de faixa etária confere à idade uma importância central, mas que pode, na realidade, ser mais bem representada por outros fatores, como a fragilidade.^(55)^ Além disso, no Brasil e em outros países do mundo, o envelhecimento é um privilégio de indivíduos de raça/cor branca e de classes sociais mais favorecidas.^(56)^

Embora diversos estudos tenham demonstrado que pessoas pretas e pardas possuem maior risco de morte associada à COVID-19,^(57-59)^ o plano de vacinação não priorizou indivíduos conforme raça/cor. Adicionalmente, indivíduos com piores condições de moradia, de saneamento básico e menor possibilidade de adesão às recomendações de distanciamento físico tampouco foram priorizados na ordem de aplicação das vacinas.

Por fim, diante do crescente movimento de hesitação com relação a vacinas,^(43)^vem sendo debatido o uso mandatório da vacina da COVID-19 em alguns contextos.^(60-63)^ Considerações éticas envolvidas nesse debate incluem a autonomia e a liberdade individual, em oposição à solidariedade e ao bem-estar coletivo. Entretanto, uma das características importantes dos direitos fundamentais é que nenhum direito é absoluto. Em outras palavras, embora a recusa à vacina seja um direito do indivíduo, essa ação pode causar prejuízos à sociedade, por exemplo, ao impedir ou atrasar benefícios coletivos, como a imunidade de rebanho e a redução da sobrecarga em serviços de saúde, ou ao colocar em risco indivíduos suscetíveis. O que deve ser compreendido é que, em uma pandemia, o interesse individual se confunde com o coletivo, pois o indivíduo apenas se protegerá quando a coletividade estiver segura. A compreensão de deveres e direitos como exercício de civismo deve ser repensada para efetivamente dar suporte ao interesse coletivo como uma extensão do interesse individual. A solidariedade se mostra relevante e precisa ser trazida à reflexão coletiva. Ninguém pode se proteger de uma epidemia individualmente. As ações de proteção ou prevenção precisam ser de natureza coletiva. Precisamos promover o equilíbrio entre a liberdade e a solidariedade social.

Políticas de uso mandatório de vacinas habitualmente contemplam isenções (por exemplo, contraindicações médicas ou restrições religiosas) e não envolvem punição direta ou ações criminais contra indivíduos, mas sim restrições em atividades, como frequentar escolas, exercer determinadas atividades profissionais^(64)^ ou realizar viagens. Para que o uso mandatório seja colocado em questão, é essencial que tenham sido esgotadas as estratégias de informação e persuasão, existam insumos suficientes para aplicação, e o uso mandatório seja corroborado proporcionalmente pelo benefício esperado (isto é, que o volume de indivíduos vacinados com essa estratégia justifique o bem coletivo atingido). É imprescindível, ainda, considerar que políticas de uso mandatório podem desencadear reações negativas, afetando a confiança da população em ações governamentais e até a adesão a outras medidas de saúde pública.^(60)^

A pandemia da COVID-19 trouxe à tona a importância da avaliação de novas tecnologias em saúde como estratégia para a implementação de novas tecnologias, como medicamentos e vacinas, na prática clínica e no SUS. Em uma pandemia marcada por elevada ocorrência de casos e óbitos e intenso impacto social, a perspectiva ética compõe e deve nortear a avaliação de aspectos clínicos, econômicos, organizacionais e sociais da avaliação de novas tecnologias em saúde. Sem o respeito e a compreensão da operação científica e da saúde, qualquer medida política está fadada ao fracasso, o que reforça a importância da avaliação de novas tecnologias em saúde. Para tanto, a solidariedade deve ser entendida como prática compartilhada que possibilita a cada um de nós assumir custos e tarefas no enfrentamento dos desafios de interesse de toda sociedade. Nesse sentido, vale lembrar que a saúde individual depende da cooperação em saúde coletiva, pautada pela transparência e pela confiança. Precisamos dar espaço para o renascimento de uma sociedade norteada pelo “nós” e não pelo “eu”.
